# Authors’ Response to Peer Reviews of “Use of Spinal Anesthesia in Pediatric Laparoscopic Appendectomies: Case Series”

**DOI:** 10.2196/29608

**Published:** 2021-04-28

**Authors:** Md Jafrul Hannan, Mosammat Kohinnor Parveen, Alak Nandy, Md Samiul Hasan

**Affiliations:** 1 Department of Pediatric Surgery South Point Hospital Chittagong Bangladesh; 2 Department of Pharmacology & Therapeutics Rangamati Medical College Rangamati Bangladesh; 3 Department of Anesthesiology Chattagram Maa-O-Shishu Hospital Medical College Chittagong Bangladesh; 4 Department of Pediatric Surgery Dhaka Shishu Hospital Dhaka Bangladesh

**Keywords:** pediatrics, appendectomy, spinal anesthesia, general anesthesia, laparoscopy, vomiting, keyhole, surgery, anesthesia, appendix

*This is the authors’ response to peer-review reports for "Use of Spinal Anesthesia in Pediatric Laparoscopic Appendectomies: Case Series."*

## Round 1

The authors of the manuscript [1] thank the editor and the reviewers [2-4] for their valuable comments and suggestions to improve the paper. We have substantially modified the manuscript to address the issues raised. We will address them individually.

### Anonymous [2]

#### Specific Comments

##### Minor Comments

1. We have changed the wording in the *Abstract* and *Introduction* sections.

2. We have addressed this and moved the descriptive statistics table to the *Results* section.

3 and 4. We have significantly modified the results and their presentation. The mosaic plots in the figures have been replaced with tables with *P* values from the Fisher exact tests for all comparisons. We have also now included odds ratios for these with upper and lower confidence levels (95%). We believe this adds to the robustness of the statistical analysis while enabling the written description of the results to follow with more brevity. We believe it is easier to read.

5. We have deleted this part as we agree it is a bit ambiguous.

### Anonymous [4]

#### General Comments

1. The groups were similar in age and gender.

2. Yes, the presence of postoperative nausea or vomiting was a binary response.

#### Specific Comments

##### Major Comments

We agree that the attempts to correlate pain scores with anesthesia were going to be confounded by the analgesics, which is why we had those figures as supplemental material. However, this was described in the manuscript proper, which we have since removed (and the supplemental figures as well).

We have left the incidence of vomiting as a measure of patient comfort in the paper. It was our goal to compare the two procedures (spinal vs general anesthesia), and by procedure, this includes the usual standard-of-care protocols for each anesthetic. Naturally, it would have been better if the exact same protocols could have been used during the administration of both anesthetics, but that is not possible. Even if the vomiting is largely a result of nitrous oxide use in the general anesthetic, that could be a good enough reason to use spinal anesthesia all by itself. Our results verify this. Additionally, there is evidence that this nitrous oxide effect is mostly predominant in longer procedures. According to Peyton and Wu [5], a “duration of exposure to nitrous oxide less than 1h has little effect on the rate of postoperative nausea and vomiting.” The maximum operation times in our study were ~45 minutes.

##### Minor Comments

1. A native English speaker has reviewed and made further copyediting changes.

2. The currency (Bangladesh taka) has now been listed in the text and along the figure axis title.

## Round 2

### Further Editorial/Peer-Reviewer Comments

The study has been labeled a “case-series report” as advised.
